# Educational interventions and communication strategies to improve HPV immunization uptake: a systematic literature review

**DOI:** 10.3389/fpubh.2025.1675946

**Published:** 2025-11-03

**Authors:** Floriana D’Ambrosio, Romina Sezzatini, Raffaella Bucciardini, Ada Maida, Anna Nisticò, Elisabetta De Vito, Walter Ricciardi, Stefania Boccia, Giovanna Elisa Calabrò

**Affiliations:** ^1^Section of Hygiene, University Department of Life Sciences and Public Health, Università Cattolica del Sacro Cuore, Rome, Italy; ^2^National Center for Global Health, Istituto Superiore di Sanità, Rome, Italy; ^3^Department of Human Sciences, Society and Health, University of Cassino and Southern Lazio, Cassino, Italy; ^4^Department of Human Sciences, Society and Health, European University of Technology EUt+, European Union, Cassino, Italy; ^5^Department of Woman and Child Health and Public Health, Fondazione Policlinico Universitario A. Gemelli IRCCS, Rome, Italy

**Keywords:** human papillomavirus, cervical cancer, HPV vaccination, educational intervention, communication strategies, healthcare professionals, vaccination coverage

## Abstract

**Introduction:**

Human Papillomavirus (HPV) infection represents a global health concern, causing approximately 627,000 cancer cases in women and 69,400 in men annually. Despite the proven value of HPV vaccines, disparities in vaccination coverage persist worldwide, highlighting the need for coordinated efforts to address vaccine acceptance and promote equitable access. To tackle this global challenge and align with the World Health Organization’s (WHO) strategy to eliminate cervical cancer by 2030, implementing effective interventions to enhance knowledge within target populations is crucial to increasing vaccination uptake. This systematic review aimed to explore educational interventions and communication strategies employed by healthcare professionals (HCPs) to improve HPV vaccine coverage.

**Methods:**

A systematic literature review was conducted by querying three databases from July 2006 to July 2025. Eligible studies were systematic literature reviews (SLRs) and primary studies not included in SLRs, focused on international educational and communication strategies implemented by HCPs targeting the WHO-recommended populations for HPV vaccination, as well as parents and other influential stakeholders involved in vaccination decision-making.

**Results:**

A total of 17 studies were included, of which 6 SLRs and 11 primary studies, with 71% (12/17) focusing on educational interventions and 29% (5/17) on communication strategies. HPV vaccine-eligible individuals were targeted in 41% (7/17) of studies, while parents and other stakeholders in 59% (10/17). Narrative videos were the most common employed strategy (53%, 9/17), followed by written informative materials (35%, 6/17), social media (29%, 5/17), and person-to-person solicitation (23%, 4/17).

**Conclusion:**

The findings underscore the importance of tailored communication strategies to raise awareness and effectively engage diverse populations. Identifying strengths and gaps in current approaches is essential for creating evidence-based interventions that not only promote reliable information but also inform effective public health policies. Aligning these efforts with the WHO’s call to action is crucial to maximizing the whole value of vaccination, reducing the global burden of HPV-related diseases, and advancing toward cervical cancer elimination by 2030.

## 1 Introduction

Human Papillomavirus (HPV) infection represents a significant worldwide health concern, contributing to a substantial burden of HPV-related diseases. Globally, approximately 627,000 cancer cases in women and 69,400 in men are attributable to HPV infections each year (1).

The role of HPV infection in the etiology of Cervical Cancer (CC), which is the most prevalent and fatal malignancy caused by the virus, is well-documented (2). Moreover, there is growing evidence of its involvement in a range of diseases affecting both men and women, including genital warts, a proportion of head and neck cancers (HNCs), anogenital cancers (anus, penis, vagina, and vulvar), and recurrent respiratory papillomatosis (RRP) (3).

Over 225 HPV subtypes have been identified, with HPV16 and HPV18 responsible for about 70% of invasive CC cases worldwide. In contrast, low-risk genotypes 6 and 11 cause around 90% of genital warts and are the main agents in RRP (4, 5).

With an estimated 662,301 new cases and 348,874 deaths in 2022, CC is the fourth leading cause of cancer among women worldwide and it is the second most common cancer in women aged 15–44 years (6).

On a global scale, the burden of CC is expected to rise further, with projections estimating 760,082 new cases and 411,035 deaths by 2030 (7).

Vaccination is the most effective prevention method for CC and other HPV-related cancers and diseases (8). Over the years, increasing scientific evidence has supported the development of bivalent, quadrivalent, and nonavalent vaccines all of which demonstrate effectiveness in preventing HPV infections and associated conditions (9). The nonavalent vaccine offers the most comprehensive protection, covering additional HPV types not included in the other vaccines (10).

Despite the available evidence, equitable global implementation of this preventive measure remains lacking, leading to significant disparities between countries (8). As of 2020, the integration of the HPV vaccination into national programs was observed in fewer than 25% of low-income and less than 30% of lower-middle-income countries (LMICs), compared to over 85% in high-income countries (11). Furthermore, 44% of the global burden of CC is in countries where girls can access HPV vaccines (12).

In response, the World Health Organization (WHO) launched a global strategy aiming to eliminate CC as a public health problem by 2030, setting ambitious vaccination and screening targets (8). Yet, persistent barriers such as low awareness, misinformation, and lack of provider recommendation continue to hinder vaccine uptake (13).

In this context, educational interventions and communication strategies play a critical role in increasing public understanding of HPV risks and the benefits of vaccination.

Healthcare professionals (HCPs), such as doctors, nurses, and other medical providers, play a vital role in HPV vaccination efforts, as they are frequently the main source of vaccine-related information. Their influence extends beyond the individuals eligible for vaccination to include parents and other key decision-makers involved in the process (14, 15). Research has consistently shown that a recommendation from a physician can significantly impact a parent’s choice to vaccinate their child (16, 17). For this reason, implementing tailored educational programs and developing clear, effective communication strategies led by HCPs is crucial to improving vaccination uptake. These efforts help increase understanding and acceptance of the vaccine, highlight the serious health risks associated with HPV, and dispel widespread myths and misinformation (18).

This systematic review, conducted within the PartnERship to Contrast HPV (PERCH) project,^1^ explored international evidence on the educational and communication approaches used by HCPs to promote HPV vaccination. By evaluating the current gaps and strengths in HPV-related knowledge and communication practices, the review aimed to support the development of effective strategies that can enable HCPs to provide accurate information on HPV prevention and help increase vaccination rates worldwide.

## 2 Methods

### 2.1 Search string

A systematic review was conducted to gather information on educational interventions and communication strategies related to HPV vaccination, implemented by HCPs for targeted populations. The review was registered in the *International Prospective Register of Systematic Review*—PROSPERO (ID: CRD420251054613), and reported in accordance with the “Preferred Reporting Items for Systematic Reviews (PRISMA)” guidelines (19). Searches were performed in PubMed, Scopus, and Web of Science (WoS) using the following keywords and synonyms: “Human papillomavirus,” “Papillomavirus,” “HPV,” “vaccination,” “vaccine,” “communication,” “healthcare workers,” “HCWs,” “health care workers,” “healthcare professionals,” “health care professionals,” “HCPs,” “medical staff,” “physicians,” “doctors,” “pediatricians,” “gynecologists,” “general practitioners,” “clinicians.” Specific search strings were tailored to each database and applied on July 24^th^, 2025.

Retrieved articles were recorded into a Microsoft Excel worksheet. After removing duplicates, the selection process followed predetermined inclusion and exclusion criteria. Initial screening was based on title and abstract, followed by a thorough evaluation of the full texts.

### 2.2 Inclusion and exclusion criteria

According to the latest WHO-recommended target population for HPV vaccination (20), all studies providing data and details on educational interventions or communication strategies implemented by HCPs, aimed at girls aged 9–14 years, females aged ≥15 years, boys, older males, men who have sex with men (MSM) and young adults, were considered potentially eligible.

For the purpose of this review, educational interventions were defined as activities primarily aimed at increasing knowledge, awareness, and skills related to HPV-vaccination (e.g., videos, training sessions), while communication strategies were defined as approaches intended to influence attitudes, perceptions or decision-making (e.g., media campaigns, posters).

Additionally, studies targeting parents, caregivers, teachers, and other key figures who could significantly influence vaccination decision-making were also considered. We included primary studies and systematic reviews conducted at international level, written in English language, and published from July 1, 2006, when the first HPV vaccine was licensed for use in adolescent girls (21). Narrative reviews, commentary, editorials, conference presentation, and references without full text, as well as studies lacking pertinent or sufficient information for the purposes of this review, were excluded.

### 2.3 Selection process and data extraction

Four researchers (F. D’A., A. M, A. N., R. S) independently screened the titles, abstracts, and full texts, resolving any disagreements through discussion or consultation with a senior researcher (G. E. C.). Additionally, a snowballing process was employed to identify further relevant papers by examining references and citations.

For each primary study, not included in the selected systematic reviews, data were extracted on first author, publication year, and country; study design; target population; characteristics of the target population (sample size, mean age, gender), along with control group details when applicable; the developer/provider of the educational intervention/communication strategies; intervention setting; utilized tools/channels; features of the educational intervention /communication strategies; and main outcomes measured.

For systematic reviews, the extracted data included the first author, publication year, country, number of studies included, target population, characteristics of the educational intervention/communication strategies, and key findings.

No predefined primary or secondary outcomes were set for this review. Instead, outcomes were extracted as reported by each study and subsequently grouped into descriptive categories: (i) HPV vaccination uptake (defined as initiation or completation of vaccination series); (ii) knowledge and awareness (awareness of HPV infection and correct understanding of HPV vaccination), (iii) attitudes and intentions toward vaccination (perceptions toward HPV vaccination and willingness to receive or recommend it), (iv) vaccine hesitancy or confidence (concerns about or trust in HPV vaccination), and (v) acceptance (agreement with HPV vaccination as a preventive measure).

### 2.4 Risk of bias assessment

The risk of bias of the included studies was assessed using validated tools, selected according to intervention design. An overall risk of bias judgment of randomized controlled trial (RCT) was elaborated using the Cochrane risk-of-bias tool (RoB2) (22).

Non-randomized intervention studies were assessed with The Risk Of Bias In Non-randomized Studies – of Interventions, Version 2 (ROBINS-I V2) (23). Cross-sectional studies were appraised using the Joanna Briggs Institute (JBI) Critical Appraisal Checklist (24), while systematic reviews were evaluated with the ROBIS tool (25).

Each study was independently assessed by four reviewers (F. D’A., A. M., A. N., R. S.), and discrepancies were resolved by discussion or by consulting a senior researcher (G. E. C.).

## 3 Results

### 3.1 Characteristics of included studies

**Figure 1:**
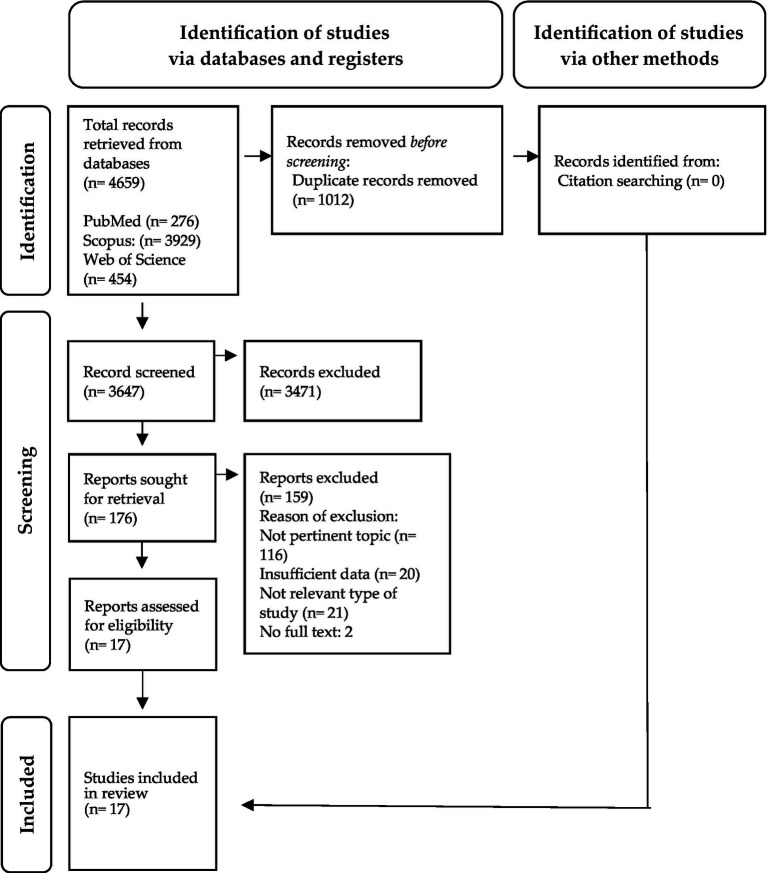
Flow chart of PRISMA study (19).

The initial database search yielded a total of 4,659 records. After removing duplicates and screening titles and abstracts, 176 full-text articles were selected for further evaluation. Following the screening process, 17 articles were included (14, 15, 22–36). The flowchart of the screening process is shown in Figure 1.

Of the 17 studies included, 11 (65%, 11/17) were primary studies (14, 15, 26–34). Among these, six (55%, 6/11) employed a non-randomized interventional design (15, 26, 28, 30, 31, 34), three (27%, 3/11) were cross-sectional studies (27, 29, 33), two (18%, 2/11) were RCT (14, 32).

Geographically, the majority of primary studies (55%, 6/11) were conducted in the USA (14, 15, 26, 31, 32, 34), two (18%, 2/11) in Italy (27, 29), and one (9%, 1/11) each in Canada (28), Africa (30), and Kenya (33).

The remaining six studies (35%, 6/17) were systematic reviews (35–40), with four (67%, 4/6) conducted at the multicountry level (35, 36, 38, 39), and two (33%, 2/6) focusing on African countries (37, 40).

In alignment with the objective of this systematic review, 12 (71%, 12/17) studies provided information on educational interventions (14, 15, 26, 28, 30, 31, 34–36, 38–40), while five (29%, 5/17) focused on communication strategies (27, 29, 32, 33, 37).

Regarding the target population, five primary studies (46%, 5/11) specifically involved populations eligible for HPV vaccination (15, 26, 28–30), of which 80% (2 /5) enrolled only females (15, 29). Three (27%, 3/11) studies targeted parents or caregivers (14, 32, 33), with one (33%, 1/3) exclusively focusing on parents of daughters (33). Additionally, three studies (27%, 3/11) included both parents and adolescents (27, 31, 34). Among the primary studies, four (36%, 4/11) also included a control group (14, 15, 31, 33).

Among the six systematic reviews, 67% (4/6) (35, 37, 38, 40) assessed interventions targeting multiple groups, including adolescents, young adults, and other relevant stakeholders such as parents, teachers, and religious leaders. In contrast, two reviews (33%, 2/6) specifically focused on adolescents and young adults aged 11–26 years (37, 39).

All interventions were developed or conducted by HCPs, with over half of the primary studies (55%, 6/11) detailing the qualifications of these professionals (14, 28, 30, 31, 33, 34). Among these, 50% (3/6) identified medical doctors as the primary developers (28, 33, 34) with a majority (67%, 2/3) involving gynecologists and oncologists (28, 34). The remaining 50% (3/6) reported the involvement of other professionals, including health educators, nurse practitioners, students, and medical assistants (14, 30, 31).

Eventually, all included studies reported at least one of the outcome categories defined in this review. The most frequently assessed outcome was attitudes and intentions toward HPV vaccination (47%, 8/17) (15, 26, 31, 33–35, 37, 39), followed by HPV vaccination uptake (35%, 6/17) (27–31, 39), HPV knowledge and awareness (29%, 5/17) (34, 35, 37, 38, 40), HPV vaccine hesitancy or confidence (18%, 3/17) (30, 32, 33), and acceptance of HPV vaccination as a preventive measure (12%, 2/17) (35, 37).

To synthesize the collected evidence, the main findings of this systematic review are presented in two dedicated sections: one focusing on educational interventions and the other on communication strategies, both organized by target population. The key characteristics of each study are summarized in Tables 1, 2.

**Table 1:** Summary of the included primary studies: main characteristics and results.

Educational intervention								
First author, publication year, Country	Study design	Target population	Characteristics of target population and/or control group (N, gender, mean age)	Developer/ provider of intervention	Setting	Tools/Channels	Characteristics of the educational intervention	Main outcome measured
Chan A. et al., 2015 (62) USA	Pre-post study	Hispanic young adults	Tot: 38 F: 31 (81.6%) M: 7 (18.4%) Age range: 18–26 years Mean age: 21.9 years	Primary care HCPs	Health Center		18-page fotonovela featuring a young Hispanic female Topics: susceptibility to disease; severity of disease; benefits of health action; barriers; self-efficacy; and cues to action	**Susceptibility and intent to HPV vaccination** Increase in attitude toward the HPV vaccine: from 71.1% at baseline to 84.2% post-intervention (*p* < 0.05) Increase in perceived susceptibility: +10.5% (*p* = 0.03) Increase in benefit of vaccination in a committed relationship: +7.8% (*p* = 0.25) Increase in intent to vaccinate: +18.4% (*p* = 0.06) Increase in intent to encourage others to vaccinate: +10.5% (*p* = 0.14)
Piedimonte S. et al., 2018 (28) Canada	Pre-post study	University students	Phase I Tot: 56 F: 43 (75.4%) M: 13 (24.6%) Mean age: 24.8 ± 7.5 years Phase II Tot: 151	Resident physicians and experts in gynecology	University campuses	Social media Person-to-person solicitation	Slideshow, on tablet: Including shocking images of CC suited for targeted population; Pamphlet distributed by medical students; Informative emails through student association newsletters; Facebook event; Educational booths on HPV and vaccination; Medical students across libraries and residences: distributing pamphlets and speaking to students.	**HPV vaccination uptake** Tot: 18 walk-ins vaccinated directly from the educational initiatives and person-to person solicitation. McGill University 2016 vs. 2015: 502 vs. 56 vaccines Concordia University 2016 vs. 2015: 455 vs. 371 vaccines
Dixon B. E. et al., 2019 (14) USA	RCT	Parents/guardians of unvaccinated or partially vaccinated adolescents aged 11–17 years old	Intervention group Subjects who received a tablet: 141 Control group: Subjects who did not receive a tablet: 1455	Medical assistants	Pediatric clinics	Video	Digital video, in English or Spanish language, based on: Reinforcement message to vaccination Information specific to the cancer prevention benefits Information specific to safety profile of the vaccine Information about the importance of receiving the full series of vaccines	**HPV vaccination attitudes** Increase in adolescents whose parents watched the video: 3-times greater odds of receiving a dose of the HPV vaccine (78.0%, *p* = 0.003).
Kim M. et al., 2019 (15) USA	Intervention study	Korean or Korean American female students aged 18–26 years old	Tot: 104 Mean age: 21.7 years Intervention group: 54 Control group: 50	Physicians	University campus		Video talking about HPV vaccine experience (17- min) Topic: storytellers of Korean female students who were born in the USA/ who moved to the USA younger than 18 years of age/who moved to the USA at age 18 or older; HCPs evidence-based information	**Susceptibility/** **Feelings about getting the HPV vaccine** Video intervention resulted in significantly greater satisfaction and more positive feelings about getting the HPV vaccine when compared with the text-based comparison group.
Drokow E. K. et al., 2021 (30) Africa	Pre-post study	Young female	Tot: 600 Age range: 19–60 years Mean age: 27 years	Health educators and nurse practitioners	Healthcare settings	Video	15-min video portrayed a pictorial illustration of CC progression and available treatment modalities.	**Awareness and HPV vaccination uptake** Capability to prevent CC and other HPV cancer types: from 25.0 to 95.0% Vaccine for males: from 18.3 to 82.5% Willingness to be vaccinated: from 47.5 to 81.7% 6 months after intervention 192 participants (32.0%) begun HPV vaccination cycle.
Santa Maria D. et al., 2021 (31) USA	Pre-post study	Parents/caregivers and their youth aged 11–14 years old	Intervention group Parents: 261 F: 234 (90.70%) M: 24 (9.30%) Youth: 255 F: 134 (53.39%) M: 117 (46.61%) Control group Parents: 258 F: 230 (89.84%) M: 26 (10.16%) Youth: 253 F: 121 (48.21%) M: 130 (51.79%)	Student nurses	n.a.	Face-to-face session	Brief face-to-face session between parents and nurses (45 min); Take-home manual; Booster calls (1- and 3-months post-intervention) All materials and sessions were available in English and Spanish.	**HPV vaccination uptake** 6 months post intervention Intervention group: 70.3% vs. Control group: 60.6% (*p* = 0.02). **Intent of parents to administer all three HPV doses** Intervention group: 72.13% vs. Control group: 54.55% (*p* = 0.0037).
Webster E. M. et al., 2024 (34) USA	Pre-post study	Parents of adolescents aged 11–17 years old Young adults aged 18–26 years old	Tot: 101 M: 12% F: 87% Mean age: 37.5 years Age range:18–62 years	Gynecologic oncologists, gynecologists, pediatricians	Pediatric clinic	Web-based audiovisual videos	PALS audiovisual modules on personal mobile device or clinic-provided tablets: to address the most common knowledge gaps and misconceptions: risks of HPV; purpose of the HPV vaccine; eligibility for the HPV vaccine; HPV vaccine side effects.	**HPV vaccine knowledge** Improvement in the post-intervention survey compared to the pre-intervention survey (score: 9.87 vs. 17.53, *p* < 0.001) **HPV vaccine attitudes** Relation with participant sex, race, ethinicity, N of children in the household, education, or religion. N
Communication strategies								
First author, publication year, Country	Study design	Target population	Characteristics of target population and/or control group (N, gender, mean age)	Developer/ provider of intervention	Setting	Tools/Channels	Characteristics of the communication strategies	Main outcome measured
Giambi C. et al., 2015 (27) Italy	Cross-sectional study	Parents, adolescents, and pre-adolescents	n.a.	HCPs of LHAs	LHAs	Brochures/leaflets (92% of LHAs); Fliers/posters (72%); LHA website (38%); Newspaper (35%); Regional Health Authority website (29%); Television (24%); Radio (15%).	Communication campaigns organized in collaboration with RHA (68%) Communication campaign coordinated at regional level (16%) Communication campaign coordinated at local level (30%) Campaigns repeated over time (30%) Translation of the informative material into other languages (13%) Communication tool: 3–6 tools (41%), <3 tools (59%) Sites of distribution of communication material Vaccination services (in 100% of LHAs); Pediatricians’ practices (75%); Women and family’s healthcare services (74%); GPs’ practices (56%); Schools (36%); Gynecologists’ practices (31%); Pharmacies (15%).	**HPV vaccination uptake** Utilizing ≥3 communication channels: ≥70%
Trucchi C. et al., 2019 (29) Italy	Cross-sectional study	Preadolescent, adults and subjects at risk	n.a.	HCPs of LHAs	Healthcare settings	-Informative material Call center Focus group Media	Informative material available at immunization centers by: Ministry of Health (7.7%); Region (42.3%); LHUs (50%); Scientific agencies (11.5%); Pharmaceutical companies (30.8%) Translation of informative material (19.2%) Call center (53.8%) Media informative campaign (34.6%) Focus group addressed to preadolescents parents (42.3%)	**HPV vaccination uptake** Communication strategies were not significantly related to vaccination coverage.
Shah P. D. et al., 2021 (32) USA	RCT	Parents of unvaccinated children aged 9–17 years old	Tot: 1196 F: 645 M:551 Mean age: 43 years	HCPs	Online	Video	Based on announcement approach: video of a pediatrician attempting to ease concerns/ or encouraging parent to get their child vaccinated.	**HPV vaccine hesitancy and confidence** Viewing video, that ease parent’s concern led to lower HPV vaccine hesitancy and higher confidence in the benefits of the HPV vaccine.
Horn S. et al., 2022 (33) Kenya (Nairobi /Nakuru)	Cross-sectional study	Parents of daughters aged 8–11 years old	Tot: 600\u00B0F: 384 M: 216 Mean age: 32.8 years Intervention group Male doctor recommendation: 200 Female doctor recommendation: 190 Control group: 210	Doctors	Online	Poster	Posters with female doctor recommendation/male doctor recommendation on HPV vaccine National campaign poster on HPV vaccination	**HPV vaccine intentions** Increase with female doctor poster: 33.7% Increase with male doctor poster: 30.5% Control group: 22.4% **Vaccine safety perceptions** Increase with female doctor poster: 24.2% Increase with male doctor poster: 28.0% Control group: 17.1%

*CC, Cervical Cancer; HCPs, Healthcare Professionals; LHAs, Local Health Authorities; N. A., Not Available; PALS, Patient Activated Learning System; Q&A, Question and answer session; RCT, Randomized Controlled Trial; RHAs, Regional Health Authorities.

**Table 2:** Summary of the included Systematic Reviews: main characteristics and results.

First author’s, publication year	Country	Number of included studies	Target population	Educational intervention and/or Communication strategies: main findings
Fu Y. L. et al., 2014 (35)	India, Hong Kong, USA, UK, Canada, Sweden, Australia	33	Adolescents, young adults, and parents	**Parental education:** Written fact sheets about HPV vaccination and potential morbidity associated with HPV infection; 1-h slide presentation about HPV infection; Spanish-language radio advertisement (*radionovela*) about HPV vaccination. **Adolescent and young adults education:** Brief HPV educational videos (3,10, 13 min), fact sheet and t-shirts; Hour-long live presentations (handouts and online resources) on HPV and condom usage delivered at school; Written HPV fact sheets, discussion of contents and reminder mailing; Online fact sheet with a question-and-answer section and a self-quiz.
Walling E. B. et al., 2016 (36)	USA, Canada, India, South Africa, Cameroon, Uganda, Rwanda, Tanzania, Australia, Brazil, Peru, Cambodia, Vietnam, Germany, England, Scotland, Switzerland, Spain, Netherlands, Italy, Denmark	51	Adolescents and young adults aged 11–26 years old	**Informational interventions parents-adolescent targeted:** Community-wide media information campaign. **Behavioral interventions parents-patients targeted:** Pamphlet emphasizing HPV CC and genital warts prevention; educational video narrated by a peer and an expert; reminder letters; text message reminders; family-focused reminders.
Oketch S. et al., 2023 (37)	Sub-Saharan Africa	22	Adolescents aged 10–19 years old, parents, caregivers, teachers, and religious leaders	**Communication strategies for vaccine acceptance:** Door-to-door communication, IEC materials, media, community meetings, face-to-face session. **Communication strategies for vaccine completation:** Community meetings, informational posters, flyers, television, radio and newspaper. **Communication strategies for knowledge, attitude, and practice**: Brochures, pamphlets, fact sheets and flyers.
Escoffery C. et al., 2023 (38)	The USA, Europa, Africa, Asia, Africa, Centro/Sud America, Canada	79	Adolescents Young adults aged 18–34 years old Parents	**Educational intervention** The most common intervention components were individual education of parents and/or adolescents (76.0%); use of technology such as websites, PowerPoints, and text messages (26.6%); and provider education (20.3%).
Sandi YDL. et al., 2024 (39)	Worldwide	12	Adolescents and young adults aged 9–26 years.old	**Educational interventions** Digital technologies, including web-based platforms, video-based content, and electronic messaging via computers or mobile phones, have been utilized in various HPV vaccination interventions. Effective strategies, with outcome measures focused on HPV knowledge, vaccine intention, and/or vaccine completion rates, included the use of email and text message reminders for appointments, videos, web-based interactive narratives, and individually tailored educational content.
Olaoye O. et al., 2024 (40)	Africa	18	Eligible individuals for the HPV vaccination and relevant stakeholders	**Educational interventions** The most common educational intervention included the use of factsheets, information leaflets, magazines, printed pamphlets, knowledge sharing events, home visits, film screening, symposia, training seminars, group-based presentations, and workshops These interventions led to increased vaccine uptake (ranging from 34 to 93.3%) and improved participants’ knowledge, attitudes, and perceptions about the vaccine. Post-intervention, there was also a high consensus on the vaccine’s safety and effectiveness, with reported agreement levels ranging from 67.9 to 90.3%.

*CC, Cervical Cancer; HPV, Human Papillomavirus; IEC, Information, Education, and Communication materials.

### 3.2 Educational interventions

Overall, our search strategy identified 12 studies (71%, 12/17) primarily aimed at evaluating the characteristics of educational interventions designed to enhance knowledge about HPV vaccination (14, 15, 22, 24, 26, 27, 30–32, 34–36). Of these, seven (58%, 7/12) were primary studies (14, 15, 22, 26, 30, 31, 34), while five (42%, 5/12) were systematic reviews (35, 36, 38–40).

Among the professionals leading these initiatives, 57% (4/7) of the primary studies reported the involvement of physicians (14, 15, 28, 34), followed by nursing staff (29%, 2/7) (30, 31), and general HCPs (14%, 1/7) (26).

Additionally, six of the seven primary studies (86%, 6/7) provided details on the context in which the interventions were implemented (14, 15, 26, 28, 30, 34). Of these, more than half (67%, 4/6) took place in healthcare settings (14, 26, 30, 34), while one-third (33%, 2/6) in university or academic environments (15, 28).

The following findings are organized according to the specific target populations for which these interventions were designed.

#### 3.2.1 Educational interventions for populations eligible for HPV vaccination

Among the 12 studies focused on educational interventions, four primary studies (33%, 4/12) (15, 26, 28, 30) and 83% (5/6) of the systematic reviews (35, 36, 38–40) described strategies aimed at enhancing knowledge about HPV vaccination among adolescents and young adults, the primary and secondary target groups for the HPV vaccination.

These strategies included various formats, such as narrative videos/storytelling, mentioned in 44% (4/9) of the articles (15, 26, 31, 36), as well as social media and person-to-person solicitations (44%, 4/9) (28, 34, 38, 40), followed by informative written fact sheets (33%, 3/9) (35, 36, 39), and slide presentations (22%, 2/9) (36, 38).

The research by Piedimonte et al. (28) underscored the value of targeted educational campaigns. One year after a previous intervention, a new initiative was launched through social media, email, information booths, and direct solicitations aimed at American students from two university campus. The combination of social media engagement, person-to-person solicitations, and the use of provocative images resulted in a twofold increase in vaccination rates compared to the previous year, with the total number of vaccinated students rising from 56 and 371 to 502 and 455, respectively (28).

Another tailored educational initiative, delivered in a narrative format, was described by Chan et al. (26). An 18-page fotonovela, available in both English and Spanish languages and centered around a young Hispanic female protagonist, was distributed at a community-based health center to promote HPV vaccine acceptance among 41 Hispanic young adults aged 18–26 years. Examining the effectiveness of this intervention, the fotonovela yielded a significant enhancement in individuals’ perceptions of their susceptibility to HPV (+10.5%, *p* = 0.03), the perceived benefits of vaccination (+7.8%, *p* = 0.25), intent to receive vaccination (+18.4%, *p* = 0.06), and intent to encourage others to vaccinate (+10.5%, *p* = 0.14). Moreover, a substantial shift in attitude toward HPV vaccination was observed, increasing from 71.1% at baseline to 84.2% post-intervention (*p* < 0.05) (26).

The remaining two primary studies (50%, 2/4) focused exclusively on video-based educational interventions specifically targeting females from specific ethnic minorities (15, 30).

Drokow et al. (30) delivered a 15-min online video to 600 Ghanaian women, explaining CC progression and HPV vaccination benefits. The intervention, led by health educators and licensed nurse practitioners, resulted in significant improvements in awareness, with the percentage of participants recognizing HPV’s protection against CC and other HPV-related diseases rising from 25.0 to 95.0%, and male vaccine eligibility increasing from 18.3 to 82.5%. By the end of the study, 32% of participants had initiated the HPV vaccination cycle.

Similarly, Kim et al. (15) implemented a cross-cultural storytelling program for 54 Korean American young women. Three peer-paired storytellers, each with different life experiences, were engaged to share their personal vaccination stories in a 17-min video. This intervention resulted in significantly higher levels of satisfaction and more positive attitudes toward receiving the HPV vaccine compared to a text-based comparison group (*n* = 50) that received written information about the vaccine (15).

These findings align with other systematic reviews included in our research, which highlighted that most educational interventions, such as written materials (e.g., brochures, fact sheets) and videos narrated by peers or experts, led to improvements in knowledge, attitudes, and perceptions about the HPV vaccine (35, 36, 40). For example, in the review by Olaoye et al. (40), post-intervention vaccine uptake ranged from 34 to 93.3%, while consensus on the vaccine’s safety and effectiveness varied from 67.9 to 90.3%. Additionally, Sandi et al. (39) emphasized the effectiveness of digital interventions delivered through web, video, or electronic platforms, noting that male participants were more likely to complete the vaccination series following these educational interventions.

#### 3.2.2 Educational interventions for parents and guardians

Out of the 12 articles examining educational interventions (14, 15, 26, 28, 30, 31, 34–36, 38, 39), three primary studies (25%, 3/12) (14, 27, 30) and four systematic reviews (67%, 4/6) (31, 33, 34, 40) focused on interventions targeting parents and/or guardians of youths eligible for the HPV vaccination. According to the included reviews (35, 37, 38, 40), the most common educational strategies for parents and guardians involved distributing written fact sheets, typically 1–2 pages in length (14, 31, 35, 38, 40). Other formats included a one-hour slide presentation on HPV infection, a radio advertisement promoting HPV vaccination (31, 35), and various handouts, posters, and websites (38, 40).

Among the primary studies, Santa Maria et al. (31) implemented an educational effort for 261 parents/caregivers and their youths (*n* = 255). This intervention consisted of a 45-min in-person session, a take-home manual, and a follow-up call. Six months later, results were compared with a control group (parents = 258; youth = 253) that had attended only the 45-min session. The intervention group showed a significantly higher intention to complete all three doses of the HPV vaccine for their child (72.13% vs. 54.55% in the control group). Additionally, 70.3% of the intervention group had initiated the HPV vaccination series, compared to 60.6% in the control group (*p* = 0.02).

More innovative approaches were explored in two studies (67%, 2/3) (14, 34).

Dixon et al. (14) implemented a digital educational intervention in the USA, using mobile tablets to deliver HPV vaccine information to 141 parents/guardians of adolescents who were either unvaccinated or only partially vaccinated. This approach led to 78% of the adolescents whose parents engaged with the tablet-based content receiving an HPV vaccine dose, compared to just 52.8% in the control group (*n* = 1455) that did not have access to the tablet-based intervention.

Lastly, Webster et al. (34) developed an online educational platform aimed at addressing low health literacy among 132 participants, including parents of children aged 11–17 years. The platform consisted of three modules designed to fill knowledge gaps about the HPV vaccination. The modules were well-received, with 89% of participants finding them enjoyable and 93% considering them easy to understand. Additionally, 90% of participants reported a better understanding of the importance of HPV vaccination. Notably, 39% of the 18 unvaccinated individuals at the start of the study received their first HPV vaccine dose within 1 month of completing the intervention.

### 3.3 Communication strategies

Nearly 29% (5/17) of the included studies, consisting of four primary studies (27, 29, 32, 33) and one systematic review (37), focused on communication strategies designed to emphasize the importance of HPV vaccination. Among the primary studies, two were executed online by HCPs (40%, 2/5) (32, 33), while the remaining were led by Local Health Authorities (LHAs) (27, 29).

Common strategies employed across these studies included community-wide media campaigns, face-to-face sessions, and community meetings (37).

The following findings, similar to those for the educational interventions, are organized according to the specific target populations addressed by each strategy.

#### 3.3.1 Communication strategies for populations eligible for HPV vaccination

Two of the five studies (40%, 2/5) examining communication strategies for HPV vaccination specifically targeted various eligible populations, including adolescents, adults, and at-risk individuals (27, 29).

Conducted in Italy, these studies focused on HPV immunization strategies implemented by LHAs (27) and regions (29). Giambi et al. (27) found that printed materials were the most common strategy among LHAs (92%) to reach adolescents and their parents. Conversely, traditional mass media platforms, such as television, radio, web, and newspapers, were employed by fewer than 50% of the LHAs surveyed. The study also highlighted that using local media and employing more than three communication channels led to a significant increase in vaccination uptake, reaching up to 70%.

Similarly, Trucchi et al. (29) identified the most commonly used regional strategies for HPV communication, including dedicated call centers for vaccine-related inquiries (53.8%), focus groups (42.3%), media campaigns (35%), and informative materials (19.2%).

#### 3.3.2 Communication strategies for parents and key stakeholders

In addition to interventions targeting HPV vaccine-eligible populations, more than half of the studies (60%, 3/5) on tailored communication strategies focused on parents and other key stakeholders involved in the vaccination decision-making process (32, 33, 37).

Shah et al. (32) assessed the impact of different provider advice using an online national sample of 1,196 parents. The study found that brief videos featuring a female pediatrician endorsing the HPV vaccination significantly increased parents’ confidence in the vaccine’s benefits and reduced vaccine hesitancy, compared to those who received general vaccination encouragement.

Similarly, Horn et al. (33) investigated the effectiveness of visual communication through a public health poster to influence decisions among 600 Kenyan parents with daughters who were eligible but not yet vaccinated for HPV. A control group (n = 210) viewed a national HPV campaign poster, while others saw an additional version that included a recommendation from either a female or male doctor. Although the results were not statistically significant, the inclusion of a doctor’s endorsement on the poster seemed to improve intentions of parents and their perceptions of the HPV vaccine’s safety.

Conversely, Oketch et al. (37) provided further insights into initiatives involving various key stakeholders, emphasizing the effectiveness of facilitating informed decision-making. This review found that efforts targeting healthcare workers and community leaders resulted in a 95% vaccination uptake rate, while interventions involving teachers and school boards led to a 92% uptake rate. In contrast, efforts aimed at policymakers were somewhat less effective, achieving an 86% uptake rate. Additionally, training programs, as well as interventions that included drama and dance, resulted in an 85% vaccination uptake rate.

### 3.4 Risk of bias results

All six non-randomized intervention studies assessed with ROBINS-I were judged at serious risk of bias, mainly due to confounding and participant selection (Supplementary Table S1) (15, 26, 28, 30, 31, 34).

The two RCTs, evaluated with RoB 2, were at low risk for randomization and missing data but raised some concerns for reporting and outcome measurement (Supplementary Table S2) (14, 32).

The three cross-sectional studies assessed with the JBI checklist were clearly described but lacked adjustment for confounders (Supplementary Table S3) (27, 29, 33).

The six systematic reviews, evaluated with the ROBIS tool, showed low risk for eligibility criteria but often unclear risk for study selection, appraisal, and synthesis (35–40). Only one review achieved an overall low risk of bias, while the others were judged as unclear (Supplementary Table S4) (39).

## 4 Discussion

This study provides a systematic review of strategic interventions aimed at increasing HPV vaccination uptake over the past 19 years, following the approval of the first HPV vaccine in 2006. During this time, an expanding body of clinical evidence has consistently demonstrated the effectiveness and safety of HPV vaccines in preventing CC and other HPV-related diseases (41).

Consequently, HPV vaccination has been progressively integrated into national immunization programs across numerous countries, with approximately 64% of nations now offering the vaccine to girls, and 24% extending coverage for boys as well (42).

Despite these advancements, CC still represents a significant public health issue, ranking as the fourth most common cause of global cancer incidence and mortality among women, and the second most prevalent malignancy in females aged 15–44 years (1).

Achieving optimal HPV vaccination coverage continues to be an ongoing challenge (8, 11), requiring global efforts to address the social, cultural, and structural barriers that hinder vaccine acceptance and equitable access (20).

Several studies have emphasized the role of knowledge gaps and insufficient information in influencing the decision-making process, particularly among adolescents and parents (43, 44).

In this context, the implementation of targeted educational interventions and communication strategies aimed at enhancing knowledge within target populations and influential figures for adolescent’s behaviors emerges as a crucial approach to improve vaccination coverage (45).

Through a comprehensive analysis of existing literature, this systematic review sought to explore the educational and communication strategies employed internationally by HCPs to increase HPV vaccine uptake, while also considering factors such as acceptability and intention.

Although the number of studies included in this review was limited, the search process provided valuable insights into the characteristics and effectiveness of interventions across different populations.

Notably, over 70% of the studies focused on educational interventions (14, 15, 26, 28, 30, 31, 34–36, 38–40), yielding promising results in enhancing knowledge, attitudes, and intentions toward HPV vaccination. These interventions were also associated with significant increases in vaccine uptake, with variations largely dependent on design, delivery mode and specific strategies employed for interventions (30, 31).

Multicomponent approaches, combining digital tools, printed materials, and in-person engagement, tended to produce stronger behavioral outcomes, such as increased vaccine uptake (ranging from 32 to 70%) (28, 30, 31). Conversely, single or low-intensity interventions, such as brief videos or short fact sheets, were more successful in improving knowledge and attitudes rather than directly influencing vaccination behaviors (14, 15, 26, 34).

Tailored interventions, including narrative storytelling (15), fotonovelas (26) and video-based approaches (14, 15, 30), were commonly reported in studies targeting adolescents and young adults, achieving particularly positive results in acceptance and intention, and highlighting how cultural relevance and emotional resonance can enhance message effectiveness (31).

In contrast, fewer studies focused on educational interventions for parents or guardians of youths eligible for the HPV vaccine (14, 31, 34). These interventions were primarily centered around written fact sheets and informational materials, with fewer instances of digital interventions (34).

Recent research has also emphasized the importance of adapting communication formats to the preferences and needs of different target groups (46). In a protocol for a digital intervention, Cordoba-Sanchez et al. (46) proposed a co-designed approach developed with input from various stakeholders. The intervention included expert-led videos for parents, interactive tools and games for adolescents, and personal testimonies intended for all audiences. This reflects a growing recognition of the value of using diverse, age-appropriate formats to enhance engagement and support informed decision-making regarding HPV vaccination.

Nevertheless, the evidence gathered emphasized the crucial role of parents’ knowledge in shaping adolescents’ vaccine acceptance and decision-making (35, 36). This aligns with other research indicating a positive correlation between favorable parental vaccine attitudes and higher vaccination rates among children (47–49).

Notably, parent-focused interventions were most effective when they combined educational content with interactive or personalized components, such as digital tools or follow-up counseling. For instance, Santa Maria et al. (31) reported that multicomponent interventions integrating face-to-face sessions, take-home materials, and reminder calls significantly improved both vaccination initiation (70.3% vs. 60.6%) and intention to complete the series (72.1% vs. 54.6%). Similarly, Dixon et al. (14) found that tablet-based educational videos increased adolescent vaccine uptake from 52.8 to 78%. These findings underscore that empowering parents through tailored, accessible, and continuous engagement is essential to strengthen vaccine confidence and supporting informed family decision-making.

Moreover, when comparing outcomes between studies targeting adolescents and those focusing on parents, it is evident that adolescents’ intentions to receive the HPV vaccine are more strongly influenced by educational initiatives. This discrepancy may be due to the settings of many adolescent focused interventions, which often took place in educational institutions where students may have been more receptive to learning about health issues (31). However, further research is needed to assess whether the positive intentions generated by these educational interventions are sustained over time and how they ultimately affect actual vaccine uptake (31).

A smaller proportion (29%) of the primary studies included in our review specifically focused on communication strategies (27, 29, 32, 33).

While educational interventions primarily aimed to improve individual knowledge and motivation, communication strategies were more focused on shaping perceptions, building trust, and supporting community-wide engagement with HPV vaccination.

Among these, visual communication approaches, including posters and videos featuring medical recommendations, demonstrated considerable potential in strengthening intentions and perceptions surrounding vaccine safety. In this context, the communication experiment conducted by Shah et al. (32) among parents of children who had not yet completed the HPV vaccine series suggested that directly addressing parental concerns can effectively reduce vaccine hesitancy while increasing motivation and confidence in its benefits. These findings suggest that effective communication must consider both how messages are delivered and what values and concerns it addresses, especially when targeting parents, who often play a decisive role in the vaccination process (50).

The collected evidence further emphasizes the importance of targeted communication strategies, highlighting the role of media campaigns, face-to-face sessions, and community-level meetings (34–36). As demonstrated by studies conducted by Giambi et al. (27) and Trucchi et al. (29), the dissemination of clear, consistent information through multiple interventions and channels plays a crucial role in the success of HPV vaccination campaigns.

Overall, communication strategies that combined multiple delivery channels and relied on trusted messengers, such as HCPs, proved more effective in enhancing vaccine confidence and uptake compared to single approaches (27, 29). Moreover, adapting messages to local contexts and cultural norms further improved audience engagement and message credibility, thereby strengthening the perceived reliability of the information provided (27, 29).

These findings highlight that the effectiveness of communication efforts largely depends on the choice of delivery modes and the perceived credibility of the messenger.

Building on this, visual and digital tools emerged as particularly powerful instruments for engaging different audiences. Among the various interventions analyzed, video-based presentations featuring visually appealing materials emerged as the most frequently used and effective channel for both educational and communication strategies, appearing in 35% of the primary studies (14, 15, 26, 30, 32, 34). Other common approaches included informative materials such as posters and brochures, social media, person-to-person solicitation, and slide presentations.

When comparing different educational interventions, video-based approaches demonstrated a particularly strong impact on HPV vaccine acceptance among both patients and their parents (14, 30, 32). Notably, interventions incorporating fotonovelas and storytelling, grounded in real-life narratives, proved to be more effective than conventional health communication materials (15, 26). These narrative-driven strategies engage audiences on both an emotional and intellectual level, helping them connect with relatable stories, and ultimately reducing resistance to health messages (48, 51).

The role of character identification in HPV-related films has also been explored (52). Frank et al. (52) found that participants who connected with specific characters perceived a higher susceptibility to the disease. Similarly, the study by Rey et al. (53) assessed the impact of HPV vaccination narratives on college-aged adults, revealing that videos featuring a mother character were the most engaging and persuasive.

Tailoring narratives to be culturally and linguistically relevant significantly boosts engagement, especially among high-risk minority groups. By incorporating culturally familiar characters and scenarios, these interventions foster deeper identification and emotional resonance, thereby enhancing their overall effectiveness (26, 51, 54).

A subset of the included studies specifically examined behavioral aspects within minority groups, including Hispanic, Korean, and African populations (15, 26, 28, 40).

Previous research has consistently identified racial disparities in HPV vaccine knowledge, underscoring the need for targeted interventions to reduce health inequities and improve population health, particularly in high-risk communities (55–57).

This issue is further reflected in the geographical distribution of our included studies, with a predominant focus on the USA (14, 15, 26, 31, 32, 34), while studies assessing the impact of HPV interventions in low- and middle-income countries remain scarce.

Notably, only four of the included studies addressed the African context (30, 33, 37, 39), despite CC being the most prevalent cancer in half of sub-Saharan African countries (58), accounting for over 120,000 cases (36). Given the high burden of HPV infection and persistently low vaccine uptake in these regions (8, 20), the lack of targeted interventions underscores the urgent need for additional initiatives aimed at addressing racial and ethnic disparities in HPV vaccination (30, 37).

This gap emphasizes the importance of developing context-specific strategies for LMICs, where limited health infrastructure and sociocultural barriers hinder vaccine implementation (8). In such settings, community-led and culturally tailored approaches, such as school-based education, peer advocacy, and the involvement of community, religious, and traditional leaders, may play a crucial role in improving awareness, accessibility, and acceptance of HPV vaccination (8).

Recent studies have reinforced this perspective. For instance, Rosser et al. (59) described community-based initiatives in LMICs that effectively reached out-of-school girls through peer tracing, churches, and local women’s groups. Similarly, Egbon et al. (60) emphasized the importance of engaging local stakeholders and community leaders to address context-specific barriers in rural Nigerian areas.

Collectively, these findings suggest that leveraging community networks and culturally adapted delivery mechanisms can enhance equity and sustainability in HPV vaccine uptake, particularly in low-resource and underserved contexts.

Thus, the enhancement of high HPV vaccination rates represents a key component of the WHO Global Strategy to accelerate the elimination of CC (8). Achieving this ambitious goal requires collaborative efforts to tackle vaccine hesitancy and ensure the dissemination of evidence-based information. Educational and communication strategies are essential components of public health (61) and should be integrated into all immunization program, addressing the specific factors contributing to vaccine hesitancy within target populations (62). Undoubtedly, HCPs, regarded as the most trusted sources of health information and vaccination guidance, remain a cornerstone in efforts to increase HPV vaccine uptake (63, 64).

Given their significant role, it is therefore necessary to develop comprehensive training programs that not only provide them with in-depth medical knowledge but also improve their communication skills (65). These training programs should equipe HCPs to deliver vaccine information in a culturally sensitive way, addressing concerns related to cultural beliefs, trust in healthcare systems, and perceived risks (66).

Additionally, more efforts are required to address prevalent misperceptions and promote a thorough understanding of the benefits of HPV vaccination, also encouraging the collaboration of various stakeholder, like teachers, educators, as well as community and religious leaders, with important role within the school and community settings (37).

Especially in priority areas for public health, like the management of HPV-related cancers, the promotion of effective collaboration and partnerships across international, national, regional, and local levels becomes essential to provide transparent and objective information to the population (67–69). Understanding the whole value of vaccination and transmit this awareness to different stakeholders is crucial for informing health policies and guiding best practices, while also countering false and misleading information (70). Thus, according to a value-based perspective, a global political commitment with health authorities, health professionals, civil society, communities, scientists, and industry represents a critical step to invest in effective communication strategies and implement high-value health care, protecting individuals by ensuring sustained high rates of vaccination coverage across all countries (70).

Despite the useful findings, there are several limitations that should be considered in our study. Firstly, only English-language articles were included, which may have limited the scope of evidence captured on this topic. Moreover, the heterogeneity among the educational and communication interventions, including variations in content, duration, delivery methods, and target populations, has limited the generalizability of the findings and made it difficult to compare outcomes. Additionally, although a formal risk of bias assessment was conducted using validated tools appropriate to each study design, variability in study quality and reporting still poses challenges for interpreting the overall strength of evidence. Finally, while we rigorously followed the PRISMA guidelines throughout the screening process, the possibility of selection bias cannot be entirely ruled out (71).

However, in our opinion, these limitations do not compromise the value of this work. In fact, our main objective was to provide a comprehensive overview of the educational interventions and communication strategies employed to inform and educate target populations about HPV vaccination.

Raising community awareness through targeted interventions and timely, comprehensive, and appropriate communication is crucial for the successful and sustainable implementation of HPV vaccination. This approach is fundamental to achieving optimal vaccination coverage (72).

## 5 Conclusion

Despite the evidence supporting the value of vaccination, CC and HPV-related diseases continue to pose significant and pressing challenges for public health.

Addressing widespread misconceptions and promoting evidence-based knowledge are crucial steps to combat the global issue of low HPV vaccine coverage.

Our study has provided valuable insights that can guide the development and evaluation of comprehensive, tailored educational and communication strategies that are essential for increasing awareness, shaping attitudes, and improving HPV vaccination coverage. However, further research is needed to refine and implement interventions that effectively enhance HPV vaccine acceptance, aligning with the ambitious goals set by the WHO for the elimination of CC and HPV-related diseases.

## Footnotes

^1^ https://www.projectperch.eu/
