# Supplementary material for: Educational interventions and communication strategies to improve HPV immunization uptake: a systematic literature review
Source: Front Public Health. 2025 Nov 3;13:1675946. doi: 10.3389/fpubh.2025.1675946 (PMC12620370; doi:10.3389/fpubh.2025.1675946)
Supplement: Supplementary file 1 [file Table_1.docx]

Supplementary Material

# Supplementary Figures and Tables

**Table S1.** Risk of bias assessments of included non-randomized studies evaluated by The Risk Of Bias In Non-randomized Studies – of Interventions, Version 2 (ROBINS-I V2).

| **First Author, Year** | **Domain 1: Confounding** | **Domain 2: Selection of participants** | **Domain 3: Classification of interventions** | **Domain 4: Deviations from intended interventions** | **Domain 5: Missing data** | | **Domain 6: Measurement of outcomes** | **Domani 7: Selection of reported result** | **Overall Risk Of Bias** |
| --- | --- | --- | --- | --- | --- | --- | --- | --- | --- |
| Chan A. et al., 2015 (26) | Serious ROB | Serious ROB | Low ROB | Low ROB | Low ROB | Moderate ROB | | Moderate ROB | *Serious* |
| Piedimonte S. et al., 2018 (28) | Serious ROB | Serious ROB | Low ROB | Low ROB | Moderate ROB | Low ROB | | Moderate ROB | *Serious* |
| Kim M. et al., 2019 (15) | Serious ROB | Moderate ROB | Low ROB | Low ROB | Low ROB | Moderate ROB | | Moderate ROB | *Serious* |
| Drokow E.K. et al., 2021 (30) | Serious ROB | Serious ROB | Low ROB | Moderate ROB | Low ROB | Moderate ROB | | Moderate ROB | *Serious* |
| Santa Maria D. et al., 2021 (31) | Serious ROB | Serious ROB | Low ROB | Moderate ROB | Moderate ROB | Low ROB | | Moderate ROB | *Serious* |
| Webster E.M. et al., 2024 (34) | Serious ROB | Serious ROB | Low ROB | Moderate ROB | Moderate ROB | Moderate ROB | | Moderate ROB | *Serious* |

D: Domain

ROB: Risk of Bias

**Table S2.** Risk of bias assessments of included RCT evaluated by The Cochrane risk-of-bias tool (RoB2)

| **First Author,**  **Year** | **D1: Randomization process** | **D2:**  **Intended Interventions** | **D3:**  **Missing data** | **D4: Measurement of the outcome** | **D5:**  **Reported results** | **Overall** |
| --- | --- | --- | --- | --- | --- | --- |
| Dixon et al., 2019 (14) | Low risk | Low risk | Low risk | Low risk | Some concerns | *Some concerns* |
| Shah et al., 2021 (32) | Low risk | Low risk | Low risk | Some concerns | Some concerns | *Some concerns* |

D: Domain

**Table S3.** Risk of bias assessments of included cross-sectional studies evaluated by the Joanna Briggs Institute (JBI) Critical Appraisal Checklist

| First author,  year |  | Q1:  Clear inclusion criteria | Q2: Population and setting described | Q3: Exposure measured validly | Q4: Outcome measured validly | Q5: Confounders identified | Q6:Strategies for confounders | Q7: Outcomes measured reliably | Q8: Appropriate statistical analysis | Overall appraisal |
| --- | --- | --- | --- | --- | --- | --- | --- | --- | --- | --- |
| Giambi et al., 2015 (27) |  | Yes | Yes | Yes | Yes | No | No | Yes | Yes | *Include* |
| Trucchi et al., 2019 |  | Yes | Yes | Yes | Yes | No | No | Yes | Yes | *Include* |
| Horn et al., 2022 |  | Yes | Yes | Yes | Yes | No | No | Yes | Yes | *Include* |

Q: question

**Table S4.** Risk of bias assessments of included systematic reviews evaluated by the ROBIS tool.

| **First Author,**  **Year** | **D1:**  **Study eligibility criteria** | **D2:**  **Identification & selection of studies** | **D3:**  **Data collection & study appraisal** | **D4:**  **Synthesis & findings** | **Overall risk of bias** |
| --- | --- | --- | --- | --- | --- |
| Fu et al., 2014 (35) | Low risk | Unclear | Unclear | Unclear | *Unclear* |
| Walling et al., 2016 (35) | Low risk | Unclear | Unclear | Unclear | *Unclear* |
| Oketch et al., 2023 (37) | Low risk | Low risk | Unclear | Unclear | *Unclear* |
| Escoffery et al., 2023 (38) | Low risk | Unclear | Unclear | Unclear | *Unclear* |
| Sandi et al., 2024 (39) | Low risk | Low risk | Low risk | Unclear | *Low risk* |
| Olaoye et al., 2024 (40) | Low risk | Low risk | Unclear | Unclear | *Unclear* |

*D: Domain
